# Shikimic acid recovers diarrhea and its complications in SD rats fed lactose diet to induce diarrhea

**DOI:** 10.1186/s42826-023-00179-y

**Published:** 2023-11-10

**Authors:** Khaled M. M. Koriem, Alaa M. A. Abdeen

**Affiliations:** 1 Department of Medical Physiology, Medical Research and Clinical Institute, National Research Centre, 33 El-Buhouth Street, Dokki, P.O. Box 12622, Giza, Egypt; 2 Department of Pathology, Faculty of Veterinary Medicine, Cairo University, P.O. Box 12211, Giza, Egypt

**Keywords:** Kidney, Sodium, Potassium, Chloride, Oxidative stress, Apoptosis

## Abstract

**Background:**

Diarrhea is the increase of excretion of human water content and an imbalance in the physiologic processes of the small and large intestine while shikimic acid is an important biochemical metabolite in plants. This study aims to study the anti-diarrheal activity of shikimic acid through restoring kidney function, antioxidant activity, inflammatory markers, sodium/potassium-ATPase activity, apoptosis genes, and histology of the kidney in SD rats fed lactose diet to induce diarrhea.

**Results:**

Thirty-six male SD rats (150 ± 10 g, 12 weeks old) were divided into 2 equal groups (18 rats/group) as follows: normal and diarrheal rats. Normal rats were divided into 3 equal groups of 6 rats each: the control, shikimic acid, and desmopressin drug groups. Diarrheal rats were also divided into 3 equal groups of 6 rats each: diarrheal**,** diarrheal rats + shikimic acid, and diarrheal rats + desmopressin drug groups. Shikimic acid restored serum urea and creatinine, urinary volume, kidney weight, sodium, potassium, and chloride balance in serum and urine. The acid returned the antioxidant (superoxide dismutase, glutathione peroxidase, catalase, malondialdehyde, NADPH oxidase activity, conjugated dienes, and oxidative index) activity and the inflammatory markers (tumor necrosis factor-α, interleukin-1β, interleukin-6, and interleukin-10) to values approaching the control values. Shikimic acid also restored the sodium/potassium-ATPase activity, the apoptosis genes *p*53 and bcl-2, and the histology of kidney tissue in diarrheal rats to be near the control group.

**Conclusions:**

Shikimic acid rescues diarrhea and its complications through restoring kidney function, serum and urinary electrolytes, antioxidant activity, inflammatory markers, sodium/potassium-ATPase activity, the apoptosis genes, and the histology of the kidney in diarrheal rats to approach the control one.

## Background

A multifactorial gastrointestinal illness called diarrhea accounts for around 5 million fatalities each year [1]. An increase in human water excretion and an imbalance in the physiological functions of the small and large intestines are the causes of diarrhea. While chronic diarrhea lasts longer than 14 days, acute diarrhea lasts for 14 days or fewer. Acute diarrhea is a symptom of acute gastroenteritis [2]. Since electrolytes are crucial for preserving the body's homeostasis and disturbances to them can result in a variety of disorders, diarrhea leads to electrolyte imbalance, also known as water-electrolyte imbalance. The most severe electrolyte alterations in this area involve aberrant sodium, potassium, and chloride levels [3, 4]. Oxidative stress and inflammatory responses are linked to diarrhea [5, 6]. Therefore, compared to control goats, newborn diarrhea goats had increased intestinal oxidative stress [7]. In diarrhea, the inflammatory indicators were elevated [8]. Acute renal damage is brought on by diarrhea [9].

An essential metabolic metabolite in plants is shikimic acid. It was initially isolated in 1885 by Johan Fredrik Eykman, whose name is derived from the Japanese blossom shikimi [10]. Shikimic acid has mostly been employed in the pharmaceutical sector to make medicines. It has antifungal, antibacterial, anti-inflammatory, and stimulating effects on hair growth qualities [11]. For the purpose of manufacturing shikimic acid on a large scale, strains that can do so are being created [12]. By boosting the pro-inflammatory and tissue-degrading states that promote skin ageing, shikimic acid shields skin cells from UV radiation, the primary driver of skin photoaging [13]. Shikimic acid is used as a therapy to protect against blood and liver toxicity [14], and to stop the advancement of osteoarthritis because it reduces cartilage deterioration, which is a primary source of cartilage pain and restricted mobility in middle-aged and older people [15]. The preventive effect of shikimic acid against cisplatin-induced kidney injury was demonstrated by Lee et al. [16] in one of the few investigations on the subject. This finding was supported by the recovery of histological kidney damage in cisplatin-treated mice. A treatment for influenza A and influenza B is shikimic acid [17]. The half-life of shikimic acid is 1.3 h, and it reached its highest level around 3 h after intragastric delivery of shikimic acid [18]. The Chinese star anise (*Illicium verum*) and the sweet-gum (*Liquidambar styraciflua*) fruit, both of which are common in North America, are sources of shikimic acid [19]. It is a characteristic of tree fern fronds known as fiddlehead [20].

Therefore, this study aims to study the anti-diarrheal activity of shikimic acid by restoring kidney function and weight, urinary volume, electrolyte balance in serum and urine, oxidative stress, inflammation, sodium/potassium-ATPase activity, apoptosis genes *p*53 and bcl-2, and the histology of kidney tissue in male SD rats fed lactose diet for 1 month to induce diarrhea.

## Results

### Physiological measures results

In comparison to the control group, diarrheal rats had significantly lower body weight, food intake, liver weight, pancreas weight, spleen weight, heart weight, and fecal pellet count but increased water intake were observed (Table 1). Throughout the study's experimental phase, there were no rat fatalities, skin patches, convulsions, or hair loss in any of the groups being examined.

**Table 1:** Effect of shikimic acid on physiological measures of normal and diarrheal rats

Parameters	Control	Shikimic acid	Desmopressin drug	Diarrheal rats	Diarrheal rats + shikimic acid	Diarrheal rats + desmopressin
Total body weight (g)	165 ± 6.24	167 ± 6.13	164 ± 6.52	128 ± 5.09^a^	163 ± 6.24^b^	162 ± 6.24^b^
Food consumption (g/day)	11.6 ± 1.2	11.4 ± 1.4	11.5 ± 1.6	7.2 ± 1.3^a^	11.3 ± 1.6^b^	11.2 ± 1.5^b^
Water intake (ml/day)	12.4 ± 1.7	12.6 ± 1.5	12.3 ± 1.2	17.8 ± 1.3^a^	12.2 ± 1.7^b^	12.1 ± 1.7^b^
Liver weight (g/100 g bw)	2.7 ± 0.08	2.6 ± 0.06	2.5 ± 0.09	1.8 ± 0.05^a^	2.4 ± 0.08^b^	2.3 ± 0.08^b^
Pancreas weight (g/100 g bw)	0.25 ± 0.03	0.27 ± 0.02	0.24 ± 0.04	0.16 ± 0.02^a^	0.23 ± 0.03^b^	0.22 ± 0.03^b^
Spleen weight (g/100 g bw)	0.34 ± 0.06	0.33 ± 0.04	0.36 ± 0.05	0.21 ± 0.03^a^	0.32 ± 0.05^b^	0.31 ± 0.07^b^
Heart weight (g/100 g bw)	0.36 ± 0.04	0.35 ± 0.06	0.38 ± 0.05	0.23 ± 0.03^a^	0.34 ± 0.03^b^	0.33 ± 0.03^b^
Fecal pellet count	38 ± 4.07	37 ± 3.86	36 ± 4.20	25 ± 3.14^a^	35 ± 4.35^b^	34 ± 4.26^b^

Number of animals = 6 rats/group. Data are represented as mean ± SEM

^a^Highly significant change compared to control

^b^Highly significant change compared to diarrheal rats

### Kidney function and weight and serum electrolytes results

Following oral administration of shikimic acid, Table 2 shows serum sodium, potassium, and chloride ions as well as serum urea, serum creatinine, and kidney weight in the normal and diarrheal groups. The information in the table makes it evident that the levels of serum sodium, potassium, and chloride ions were much lower. (*P* ≤ 0.01) but serum urea and creatinine were significantly increased (*P* ≤ 0.01) while kidney weight was significantly decreased (*P* ≤ 0.01) in diarrheal group. All the aforementioned measures were brought close to the control levels by the oral treatment of shikimic acid to diarrheal rats. On the other hand, normal rats given oral doses of shikimic acid or desmopressin did not show any alteration during the study's experimental phase.

**Table 2:** Effect of shikimic acid on kidney function, electrolytes, and weight in normal and diarrheal rats

Groups	Serum electrolytes levels		Serum urea and creatinine levels		Kidney weight (g/100 g bwt)	Serum chloride (mmol/L)
	Sodium (mmol/L)	Potassium (mmol/L)	Urea (mg/dL)	Creatinine (mg/dL)		
Control	152.32 ± 4.64	5.58 ± 0.26	24.81 ± 2.71	0.82 ± 0.07	0.69 ± 0.05	104.29 ± 2.35
Shikimic acid	150.46 ± 3.85	5.56 ± 0.31	24.75 ± 2.39	0.81 ± 0.05	0.70 ± 0.07	102.87 ± 2.46
Desmopressin drug	151.71 ± 4.53	5.59 ± 0.27	24.84 ± 2.80	0.80 ± 0.06	0.67 ± 0.09	105.12 ± 2.18
Diarrheal rats	98.76 ± 4.03^b^	2.52 ± 0.34^b^	41.20 ± 2.67^b^	1.43 ± 0.05^b^	0.48 ± 0.03^b^	79.61 ± 1.65^b^
Diarrheal rats + Shikimic acid	145.8 ± 3.75^c^	5.19 ± 0.24^c^	29.74 ± 2.65^c^	0.79 ± 0.06^d^	0.67 ± 0.07^d^	98..50 ± 2.41^c^
Diarrheal rats + Desmopressin drug	151.24 ± 3.50^d^	5.49 ± 0.32^d^	25.26 ± 3.59^d^	0.81 ± 0.04^d^	0.71 ± 0.06^d^	101.54 ± 1.86^d^

Number of animals = 6 rats/group. Results were expressed as mean ± SEM. Statistical analysis was one-way ANOVA test

^a^*P* ≤ 0.05 significant change compared to control group

^b^*P* ≤ 0.01 highly significant change compared to control group

^c^*P* ≤ 0.05 significant change compared to diarrheal group

^d^*P* ≤ 0.01 highly significant change compared to diarrheal group

### Urinary volume and electrolytes results

Following oral administration of shikimic acid, Table 3 displays urine volume and urinary sodium, potassium, and chloride ions in the normal and diarrheal groups. The data in this table refers to significant decrease (*P* ≤ 0.01) in urinary volume and urinary sodium, potassium, and chloride ions but a significant increase (*P* ≤ 0.01) in urinary volume was observed in diarrheal rats. All the aforementioned parameters were brought closer to the control values when rats with diarrhea were given either the medicine shikimic acid or the drug desmopressin orally. Additionally, normal rats given oral doses of shikimic acid or desmopressin did not exhibit any changes during the duration of the trial.

**Table 3:** Effect of shikimic acid on urinary volume and electrolyte excretion in normal and diarrheal rats

Group	Urinary volume (mL/100 g/8 h)	Diuretic index	Urinary Sod. (mmol/L)	Urinary Pot. (mmol/L)	Saluretic index		Urinary Sod./Pot	Urinary Chlor
					Sod.	Pot		
Control	0.96 ± 0.19	–	98.52 ± 6.28	56.14 ± 2.90	–	–	1.75	7.61 ± 0.45
Shikimic acid	0.94 ± 0.23	–	96.94 ± 5.73	55.68 ± 2.83	–	–	1.74	7.59 ± 0.58
Desmopressin drug	0.97 ± 0.18	–	97.64 ± 5.48	57.14 ± 2.69	–	–	1.70	7.60 ± 0.39
Diarrheal rats	1.30 ± 0.41^b^	1.35	130.41 ± 7.20^b^	75.65 ± 3.12^b^	1.32	1.35	1.72	5.28 ± 0.27^b^
Diarrheal rats + shikimic acid	1.04 ± 0.20^c^	1.08	103.74 ± 5.39^c^	60.13 ± 2.79^d^	1.05	1.07	1.73	6.87 ± 0.59^c^
Diarrheal rats + desmopressin drug	1.01 ± 0.36^c^	1.05	101.26 ± 4.80^d^	59.07 ± 2.24^d^	1.03	1.05	1.71	6.98 ± 0.43^c^

Number of animals = 6 rats/group. Results were expressed as mean ± SEM. Statistical analysis was one-way ANOVA test

^a^*P* ≤ 0.05 significant change compared to control group

^b^*P* ≤ 0.01 highly significant change compared to control group

^c^*P* ≤ 0.05 significant change compared to diarrheal group

^d^*P* ≤ 0.01 highly significant change compared to diarrheal group. Diuretic index: Urine volume of treated group/Urine volume of the control group. Saluretic Sodium/Potassium index: Urinary Sodium/Potassium of treated group/Urinary Sodium/Potassium of the control group. Sod.: Sodium. Pot.: Potassium. Chor.: Chloride

### Kidney antioxidants results

The impact of shikimic acid on antioxidant levels in the kidney of the normal and diarrheal groups is shown in Table 4. It is obvious that diarrhea induced a highly significant decrease (*P* ≤ 0.01) in superoxide dismutase, glutathione peroxidase, catalase activities, and NADPH oxidase activity compared to the control group but a highly significant increase (*P* ≤ 0.01) in malondialdehyde, conjugated dienes, and oxidative index respectively, compared to the control group. Additionally, when given orally to diarrheal rats, shikimic acid or the medication desmopressin caused the results of the aforementioned antioxidant tests to approach control values. Furthermore, normal rats given oral doses of either shikimic acid or the desmopressin medication showed no change in any of the antioxidants used.

**Table 4:** Effect of shikimic acid on antioxidants levels in the kidney of normal and diarrheal rats

Parameters	Control	Shikimic acid	Desmopressin drug	diarrheal rats	diarrheal rats + shikimic acid	diarrheal rats + desmopressin
Superoxide dismutase (U/g tissue)	3250 ± 60	3240 ± 50	3245 ± 55	1170 ± 40^b^	3230 ± 50^d^	3220 ± 70^d^
Glutathione peroxidase (U/g tissue)	785 ± 19	780 ± 24	785 ± 21	365 ± 18^b^	775 ± 16^d^	770 ± 21^d^
Catalase (µmol H_2_O_2_/ min/mg tissue)	0.18 ± 0.05	0.19 ± 0.04	0.17 ± 0.06	0.09 ± 0.03^b^	0.14 ± 0.06^c^	0.15 ± 0.05^c^
Malondialdehyde (µmol/g tissue)	8.52 ± 0.60	8.49 ± 0.72	8.50 ± 0.69	19.38 ± 0.54^b^	9.54 ± 0.83^d^	9.58 ± 0.90^d^
NADPH oxidase activity (mg/mg protein × 10^5^)	12.3 ± 1.18	12.5 ± 1.54	12.4 ± 1.37	8.6 ± 1.31^a^	10.5 ± 1.25^c^	10.7 ± 1.26^c^
Conjugated dienes (μmol/g tissue)	1.45 ± 0.19	1.42 ± 0.17	1.43 ± 0.15	1.86 ± 0.24^a^	1.64 ± 0.19^c^	1.62 ± 0.17^c^
Oxidative index (A_233_/A_215_ ratio)	0.49 ± 0.03	0.47 ± 0.05	0.50 ± 0.03	0.62 ± 0.04^a^	0.54 ± 0.05^c^	0.52 ± 0.06^c^

Number of animals = 6 rats/group. Results were expressed as mean ± SEM. Statistical analysis was one-way ANOVA test

^a^*P* ≤ 0.05 significant change compared to control group

^b^*P* ≤ 0.01 highly significant change compared to control group

^c^*P* ≤ 0.05 significant change compared to diarrheal group

^d^*P* ≤ 0.01 highly significant change compared to diarrheal group

### Kidney inflammatory markers results

Shikimic acid's impact on inflammatory markers in the kidneys of normal and diarrheal groups is seen in Table 5. It can be estimated that diarrhea induced a highly significant increase (*P* ≤ 0.01) in tumor necrosis factor-α, interleukin-1β, and interleukin-6 levels but a highly significant decrease (*P* ≤ 0.01) in interleukin-10 level compared to the control group. As opposed to diarrheal rats, oral administration of the drugs shikimic acid or desmopressin caused the inflammatory markers listed above to approach control values. Additionally, neither oral administration of the desmopressin medication nor shikimic acid to normal rats changed any of the inflammatory markers found.

**Table 5:** Effect of shikimic acid on inflammatory markers in the kidney of normal and diarrheal rats

Parameters	Control	Shikimic acid	Desmopressin drug	diarrheal rats	diarrheal rats + shikimic acid	diarrheal rats + desmopressin drug
Tumor necrosis factor-α (ng/g tissue)	46.8 ± 2.71	45.9 ± 2.68	47.3 ± 2.59	65.2 ± 2.59^a^	45.3 ± 2.64^d^	45.6 ± 2.48^d^
Interleukin-1β (ng/g tissue)	6.5 ± 0.4	6.4 ± 0.7	6.6 ± 0.5	8.6 ± 0.3^a^	8.4 ± 0.6^c^	8.7 ± 0.5^c^
Interleukin-6 (pg/g tissue)	54.6 ± 5.7	55.2 ± 6.1	53.8 ± 4.9	107 ± 4.5^b^	56.1 ± 7.2^d^	57.0 ± 6.3^d^
Interleukin-10 (pg/g tissue)	36.4 ± 3.9	34.8 ± 3.7	35.7 ± 4.0	24.5 ± 2.3^a^	33.8 ± 3.6^c^	32.9 ± 4.1^c^

Number of animals = 6 rats/group. Results were expressed as mean ± SEM. Statistical analysis was one-way ANOVA test

^a^*P* ≤ 0.05 significant change compared to control group

^b^*P* ≤ 0.01 highly significant change compared to control group

^c^*P* ≤ 0.05 significant change compared to diarrheal group

^d^*P* ≤ 0.01 highly significant change compared to diarrheal group

### Kidney sodium/potassium-ATPase results

**Fig. 1:**
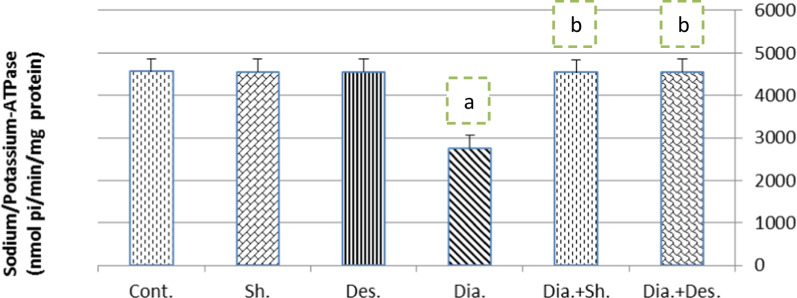
Effect of shikimic acid and on Sodium/Potassium-ATPase activity in the kidney of normal and diarrheal rats. Cont.: Control rats. Sh.: Shikimic acid group. Des.: Desmopressin drug group. Dia.: Diarrheal rats. Dia. + Sh.: Diarrheal rats after oral administration with shikimic acid. Dia. + Des.: Diarrheal rats after oral administration with desmopressin drug. Number of animals = 6 rats/group. Data are represented as mean ± SEM. ^a^ Significant change compared to control. ^b^ Significant change compared to diarrheal rats

The impact of shikimic acid on sodium/potassium-ATPase activity in the kidney of the normal and diarrheal groups is shown in Fig. 1. It is clear from this figure that diarrhea induced a highly significant decrease (*P* ≤ 0.01) in sodium/potassium-ATPase activity compared to the control group. Furthermore, when given orally to diarrheal rats, shikimic acid or the medication desmopressin caused the sodium/potassium-ATPase activity indicated above to approach control values as opposed to the diarrheal rats. Additionally, during the study period, normal rats that were given shikimic acid or desmopressin orally did not have significant changes in sodium/potassium-ATPase activity.

### Kidney apoptosis genes *p*53 and bcl-2 results

**Fig. 2:**
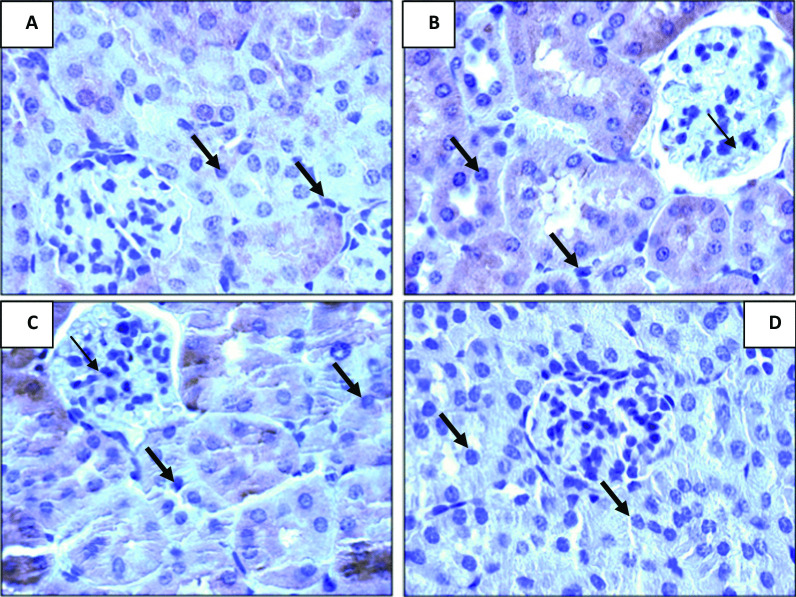
Effect of shikimic acid on *p*53 gene in the kidney of normal and diarrheal rats. **A** Immuno-histochemically stained kidney tissue reveals tumor suppressive gene *p*53 expression in the control group, showing a normal amount of expressed tumor suppressive gene* p*53. **B.** Kidney tissue of diarrheal rats reveals a decrease in *p*53 expression, which appeared as weak immune-staining tissue. **C**. An increase in expression of tumor suppressive gene *p*53 in the kidney of diarrheal rats treated with desmopressin drug appeared in the positive staining kidney tissue. **D**. The expression of the tumor suppressive gene* p*53 was increased in diarrheal rats after oral administration of shikimic acid, where a positive staining of kidney tissue was observed

According to Fig. 2, immunohistochemically stained kidney tissue from the control group displayed normal levels of expressed tumour suppressive gene *p*53 (Fig. 1A), but the amount of expressed *p*53 was significantly lower in diarrheal rats (Fig. 1B) compared to control rats, and when shikimic acid was given orally to diarrheal rats, the amount of expressed *p*53 was increased to levels that were closer to control (Fig. 1C and D) than in control rats.

**Fig. 3:**
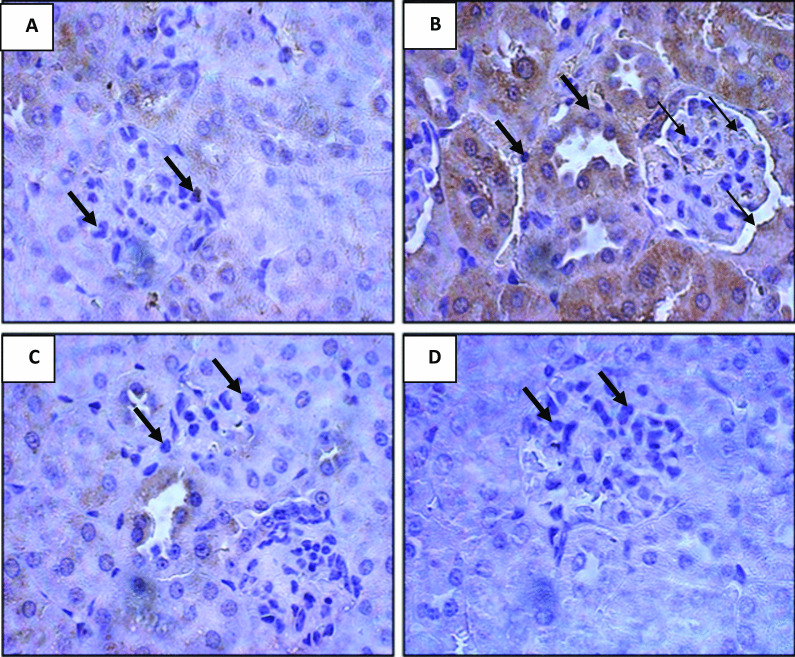
Effect of shikimic acid on bcl-2 gene in the kidney of normal and diarrheal rats. **A** Immunohistochemically stained kidney tissue, shows bcl-2 gene expression in the control group, which is characterized by a normal amount of expressed bcl-2 gene. **B** Diarrheal rats kidney tissue shows an increase in bcl-2 gene expression, which appeared as a strong immune staining. **C** Shows diarrheal rats after oral administration of the desmopressin drug, which appeared as a decrease in bcl-2 gene expression in the kidney tissue, which appeared as weak immune staining. **D** Diarrheal rats after oral administration of shikimic acid exhibit a little bcl-2 expression, which appeared as a rarer immune staining

Kidney tissue stained with immunohistochemistry is shown in Fig. 3. In contrast to the diarrheal group, the normal rats showed a normal amount of expressed bcl-2 gene (Fig. 2A), whereas the amount of bcl-2 gene was significantly increased in diarrheal rats (Fig. 2B), and significantly decreased in diarrheal rats after oral administration of shikimic acid (Fig. 2C, D).

### Histology results

**Fig. 4:**
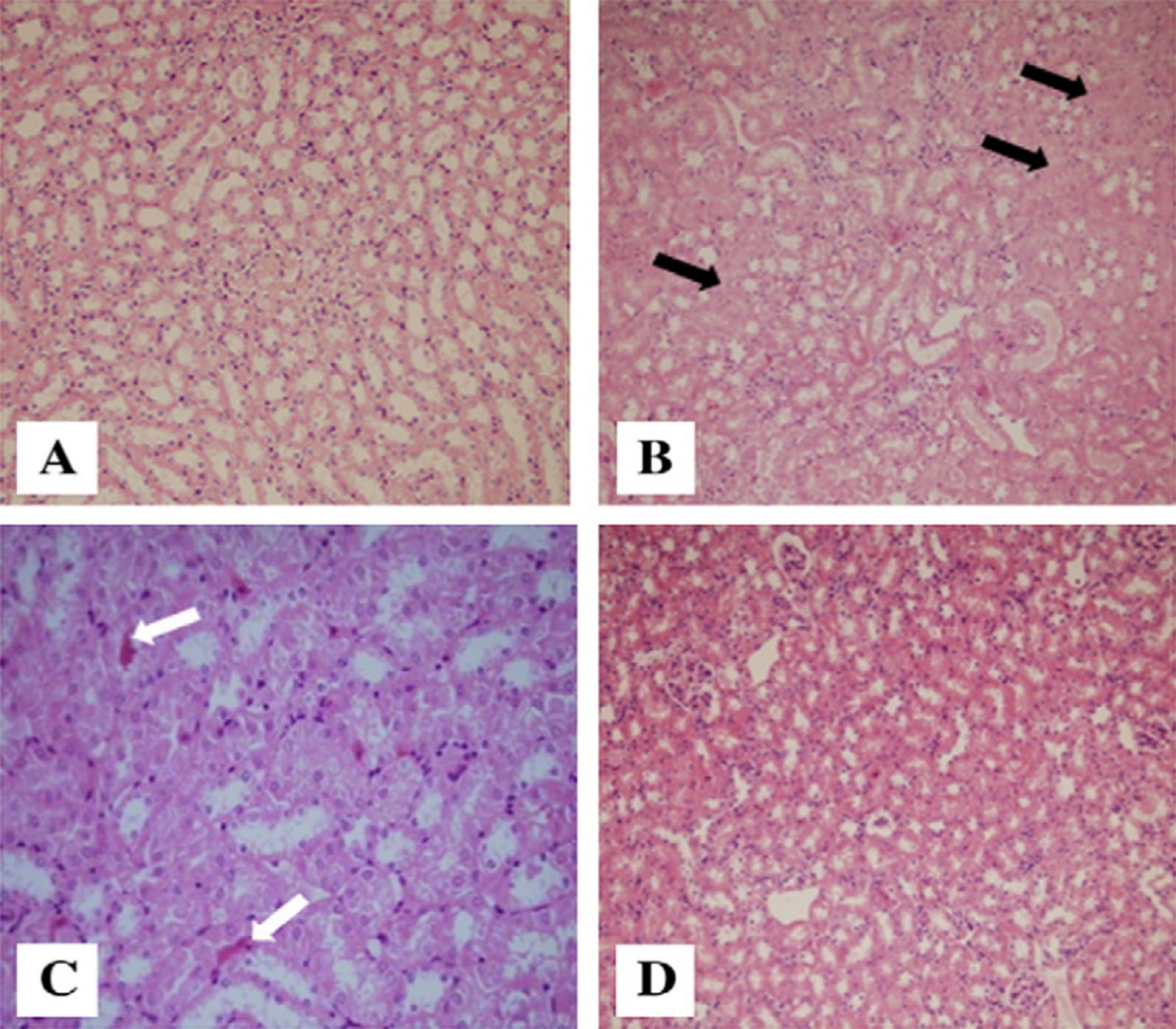
Effect of shikimic acid on kidney tissue in normal and diarrheal groups. A photomicrograph of rat kidney (H&E) from: (**A**, 200) The control group reveals normal kidney construction; (**B**, 200) and (**C**, 400) the diarrheal group treated with shikimic acid exhibits widespread coagulative necrosis (**B**, black arrows) with dilatation, vacuolar degeneration, epithelial desquamation, and intraluminal cast formation (**C**, white arrows) in the proximal tubules; (**D**, 200) The diarrheal group after oral administration with shikimic acid shows an obvious improvement in the histological construction and looks like the control group

The control group with normal renal development is shown in Fig. 4 (Fig. 3A). However, diarrhea led to a clear and widespread necrosis. Additionally, it led to vacuolar degeneration and dilatation. Particularly in the proximal tubules of the kidney, it caused intraluminal group creation and epithelial desquamation (Fig. 3B). In groups that received shikimic acid and desmopressin orally, the kidney tissue damage brought on by diarrhea was totally reversed, and the groups resembled the control group (Fig. 3C and D).

## Discussion

According to the current study, compared to the control group, diarrhea significantly raised serum urea and creatinine levels. Furthermore, it caused a highly significant increase in urine volume and urinary electrolyte excretion but a highly significant decrease in kidney weight and serum electrolytes. However, oral shikimic acid delivery to diarrheal rats caused all of the aforementioned parameters to return to normal ranges. This action is connected to shikimic acid's nephro-protective properties, which the substance demonstrated in studies against cisplatin-induced renal injury [21]. On the other hand, desmopressin treatment prevented chronic hyponatremia by preventing excessive urinary water losses, therefore hyponatremia or a drop in serum sodium did not occur in desmopressin-treated rats because desmopressin and hypertonic saline correct severe hyponatremia [22]. Desmopressin reduces the rate of change of plasma sodium and corrects hyponatremia [23, 24]. Additionally, shikimic acid increased the mRNA expression of keratinocyte growth factor, vascular endothelial growth factor, and insulin-like growth factor-1 (IGF-1) in the hair follicles [25]. Anabolic growth hormone IGF-1 is in charge of cell division, proliferation, and survival. According to the study, IGF-1 promotes sodium/potassium-ATPase [26], which results in a modest rise in serum sodium in rats given with shikimic acid as opposed to rats treated with desmopressin.

In the kidneys of diarrheal rats, where inflammation and oxidative state are associated with diarrhea [27], diarrhea generated oxidative stress. To restore and boost the antioxidant enzyme activity in the kidneys of diarrheal rats, shikimic acid was given orally. The antioxidant and anti-inflammatory properties of shikimic acid are connected to this phenomenon. Numerous earlier investigations, such as Sun et al.’s [28] claim that shikimic acid has anti-inflammatory, analgesic, and antioxidant effects, support such a conclusion. Additionally, Xing et al. [29] reported that shikimic acid has an anti-inflammatory effect, and that treatment with the acid for 6 days at doses of 100 or 200 mg/kg was able to completely protect rats from developing colitis after being exposed to acetic acid. This protective effect was attributed to the acid's antioxidant activity. Additionally, Rabelo et al.’s [30] research demonstrated that shikimic acid has anti-inflammatory properties that can be used to treat painful and pro-inflammatory disorders. Furthermore, Chang et al. [31] discovered that shikimic acid exhibited substantial antioxidant activity by inhibiting peroxidation, with activity superior to that of -tocopherol and comparable to that of tert-butylhydroquinone, propyl gallate, and gallic acid. Additionally, shikimic acid has been shown by Rabelo et al. [32] to have neuro-protective effects against oxidative stress-induced toxicity on human neuronal-like cells, suggesting that this acid may be used in the treatment of neurodegenerative illnesses that are linked to oxidative stress.

The sodium/potassium pump was out of balance because diarrhea increased the excretion of sodium, potassium, and chloride ions in the urine while depleting these ions in the serum. As a result, diarrhea decreased sodium/potassium-ATPase activity in the kidney of diarrheal rats. The sodium–potassium ATPase pump controls the balance between sodium and potassium by pumping sodium out of the cells in return for potassium moving into the cells [33]. The primary enzyme that propels trans-epithelial ionic transport is the sodium–potassium ATPase, and diarrhea lowers this enzyme’s activity [34]. Rats with diarrhea who were given shikimic acid orally had altered sodium/potassium-ATPase activity in their kidneys. This conclusion is connected to the observation that shikimic acid has antioxidant and anti-inflammatory effects by scavenging the superoxide radical and hydroxyl radical, and as a result, has analgesic, anti-inflammatory, and antioxidant activities [35]. Additionally, due to its anti-oxidation mechanism, suppression of arachidonic acid metabolism, and modification of the inhibitor kappa B-alpha/nuclear factor kappa B *p*65 expression, shikimic acid has considerable therapeutic benefits on experimental colitis in rats [36].

Shikimic acid inhibits apoptosis, and the *p*53 and bcl-2 genes showed this inhibitory impact. While the bcl-2 gene is referred to as a tumor-stimulating gene, the *p*53 gene is referred to as a tumor-suppressive gene. Cancer and toxicity are linked to both genes [37]. One of the main initiators of apoptosis in many human and animal cells is the cancer suppressive gene *p*53. By speeding up the transcription of numerous downstream genes like *p*21 and Bax to trigger an apoptotic process, it accelerates various signals that work through extrinsic and intrinsic means of apoptosis [38] and subsequently prevents the proliferation of cells with DNA damage. However, the bcl-2 gene inhibits the apoptotic process in numerous ways. The effect of pro-apoptosis is neutralized as a result of the bcl-2 gene's heterodimer complex formation with the Bax gene [39]. While diarrhea dramatically elevated bcl-2 expression, it significantly lowered the expression of the tumor-suppressing gene *p*53. Additionally, oral feeding of shikimic acid to rats with diarrhea returned the expression of the tumor-suppressing gene *p*53 and the tumor-stimulating gene bcl-2 to control levels. This outcome was consistent with Tang et al.'s [40] findings, which claimed that treatment with shikimic acid lowers apoptosis and reactive oxygen species over-generation brought on by ischemic injury, and as a result, treatment with shikimic acid has a protective effect on the brain under ischemic conditions. Shikimic acid has a protective effect against cisplatin-mediated kidney injury, as well, according to Lee [16], and this finding was supported by the recovery of histological renal injury in mice after cisplatin treatment. Furthermore, Manna et al. [41] demonstrated how shikimic acid restored H_2_O_2_-induced oxidative damage in hepatocytes by reducing apoptosis.

Shikimic acid had a cytoprotective impact on the kidney of diarrheal rats, according to histopathological analysis. This effect was connected to the acid's antioxidant and anti-inflammatory properties. Zhang et al.'s findings [42] that shikimic acid improves the histology and many serological measures, where the acid mitigates kidney damage brought on by hyperuricemia in mice, support this observation. In rats with diarrhea, there is an increase in the breakdown of serum protein and, as a result, an increase in the amount of protein excreted in the urine [43]. On the other hand, shikimic acid works as a significant therapeutic agent in the treatment of osteoarthritis by halting the breakdown of type II collagen caused by tumor necrosis factor (TNF). In human primary chondrocytes cultured in vitro, shikimic acid decreased TNF-induced production of matrix metalloproteinase 1, 3, and 13, and enhanced expression of tissue inhibitor of metalloproteinase 1 and 2 [44].

## Conclusions

By improving kidney function, urinary volume, kidney weight, electrolyte balance in serum and urine, oxidative stress, inflammation, sodium/potassium-ATPase activity, the apoptosis genes *p*53 and bcl-2, and the histology of kidney tissue in diarrheal rats, shikimic acid demonstrated anti-diarrheal effect. It will be fascinating to examine whether shikimic acid can reverse the diarrhea-related symptoms, as in real-life circumstances, in this investigation, which will be carried out as a clinical trial in diarrheal patients to determine its effectiveness.

## Methods

### Experimental animals and design

Thirty-six male albino *Sprague–Dawley* (SD, 150 ± 10 g, 12 weeks old) strain rats were purchased from the National Research Centre's animal house in Dokki, Giza, Egypt. The experimental plastic cages held the animals. They were kept alive by drinking tap water and eating a commercially balanced meal. The investigation was carried out in accordance with the proper handling and use of the laboratory animals as described in NIH publication no. 85:23, updated 1985, and after gaining clearance from the ethics committee of the National Research Centre, Egypt (approved number 12031106). The animals were divided into 6 equal groups (6 rats/group) as follows: *Control group*: normal rats were administered orally with 1 mL of distilled water. *Shikimic acid-treated group*: normal rats were administered orally with 200 mg/kg of shikimic acid [28] dissolved in 1 mL distilled water. This dose was found to be effective as a sulfasalazine (500 mg/kg) drug [45]. *Desmopressin-treated group*: normal rats were administered orally with 1 mg/kg of the desmopressin drug [46] dissolved in 1 mL distilled water. *Diarrheal group*: diarrheal rats were administered orally with 1 mL of distilled water. *Diarrheal group + shikimic acid-treated group*: diarrheal rats were administered orally with shikimic *acid (200 mg/kg)* dissolved in 1 mL distilled water. *Diarrheal group + desmopressin drug-treated group*: diarrheal rats were administered orally with 1 mg/kg of desmopressin drug dissolved in 1 mL distilled water.

All the above-mentioned treatments were administered once a day by oral gavage for 4 weeks. The animals were observed carefully during the whole study period for any abnormal physical and clinical signs such as loss of rat hair, appearance of skin patches, rat convulsions, and any deaths during the experimental study.

### Induction and assessment of diuretic activity

Rats with and without diarrhea were separated into two equal groups of the creatures. The rats with diarrhea were fed a meal containing lactose for a month in order to cause diarrhea, whereas the healthy animals were fed a typical rat diet. The diuretic activity of the animals was discovered using the approach used by Kau et al. [47]. One rat was kept in the metabolic cage for a full day prior to the commencement of the study on how animals adapt to their surroundings. All of the rats received the physiological saline (0.9% NaCl) orally prior to the experiment. Urinary volume was gathered and assessed after the research. Additionally, the levels of urine electrolytes such sodium, potassium, and chloride ions were measured as mmol/L.

## Experimental procedure

### Serum collection

Through the retro-orbital plexuses, capillary tubes containing ethylenediamine tetraacetic acid were used to acquire and collect blood samples. Centrifugation was used to produce the serum (6000 g at 4 °C), which was then frozen at − 80 °C deep freezer. The amounts of sodium, potassium, and chloride ions in the serum and urine were measured using the techniques described by Jooste and Strydom [48], Wang et al. [49], and Hassan et al. [50], in that order. According to the protocols outlined by Orsonneau et al. [51] and Myre et al. [52], serum urea and creatinine were determined. Following the manufacturer's instructions, commercial kits were used to perform spectrophotometric detection of all the aforementioned parameters.

### Kidney tissue preparation

All of the rats were given a diethyl ether solution inhalation anesthesia at the conclusion of the experimental investigation. They were dissected after being beheaded. After being obtained, the kidney tissue was cleaned in a saline solution. The filter papers were used to dry this tissue. After being separated into two pieces, the kidney tissue was first dissolved in 2.5 ml of Tris buffer solution. The tissue was then homogenized for 10 min at room temperature in an automated homogenizer. The supernatant, which was used to calculate the biochemical parameters, was removed from the tissue by centrifuging it for 15 min at − 4 °C and 7000 rpm. The second kidney tissue section was used for histological analysis.

### Biochemical analysis

NADPH, ethylene glycol-bis(2-amino-ethylether)-*N*,*N*,*N*′,*N*′-tetraacetic acid (EGTA), lubrol, potassium phosphate monobasic (KH_2_PO_4_), and lucigenin (9, 9′-bis[*N*-methyl acridinium nitrate) were all purchased from Sigma-Aldrich Company in the United States. Desmopressin (1-deamino-8-d-arginine vasopressin, ddAVP) was used as a common antidiuretic medication and was purchased from El-Kahera Pharmaceutical Industrial Company in Egypt. Through a local Egyptian supplier, all kits and reagents utilized in this study for biochemical analysis were obtained from Bio-diagnostics Company, United Kingdom.

### Kidney antioxidants detection

The Suttle method [53] was used to measure the activity of the enzyme superoxide dismutase (SOD). The Pagalia and Valentine [54] approach was used to identify glutathione peroxidase (GPx) activity. The Aebi [55] technique was used to measure the catalase (CAT) activity. Malondialdehyde levels were also determined using the Okhawa et al. [56] method as a sign of lipid peroxidation. Spectrophotometric measurements of each of the aforementioned antioxidants were made using commercial kits in accordance with the manufacturer's instructions.

According to Kogure et al.’s method [57], conjugated dienes (CD) were made by adding kidney homogenate (0.010 mg of protein) to 1 mL of 10 mmol/L phosphate buffer (pH 7.4) with 1% Lubrol. Using a spectrophotometer, the absorbance ratio A233/A215 (oxidative index) was used to calculate the production of conjugated dienes [58, 59].

A lucigenin-enhanced chemiluminescence test was used to measure NADPH oxidase activity in order to assess NADPH oxidase-mediated superoxide radical (O_2_^−^) generation [60]. Lucigenin (5 mol/L) was added after NADPH (0.1 mmol/L) was added to kidney homogenate (250 µL), which already included phosphate buffer (50 mmol/L KH_2_PO_4_, 1 mmol/L EGTA, 150 mmol/L sucrose, pH 7.4). A Tecan Infinite M200 multimode microplate fluorometer was used to measure the luminescence at 30 °C every 5 s for 10 min. NADPH oxidase activity was reported as mg/mg protein [61].

### Kidney inflammatory markers determination

According to the Matalka et al. [62] technique, tumour necrosis factor-α (TNF-α) was assessed. The DeCicco et al. method [63] was used to determine the interleukin-1β (IL-1β) level. In order to measure interleukin-6 (IL-6) and interleukin-10 (IL-10), Stelmasiak et al.’s [64] method was used. ELISA kits were used to measure all inflammatory markers while adhering to the manufacturer's guidelines.

### Kidney sodium/potassium-ATPase determination

A solution containing 80 mM NaCl, 20 mM KCl, 5 mM MgCl_2_, 50 mM Tris HCl (pH 7.4), and 3 mM ATP disodium salt was made in order to identify sodium/potassium-ATPase activity. The reaction was subsequently stopped by adding 50 mL of trichloroacetic acid after 10 min of incubation at 37 °C with 50 mL of homogenate. The mixture was centrifuged for 15 min at 3000 rpm. 250 mL of ammonium molybdate, 500 mL of trichloroacetic acid, and 250 mL of ascorbic acid were combined with 1 mL of the supernatant. A spectrophotometer was used to measure the resulting colour at 680 nm [65].

## Molecular analysis

### Immunohistochemical detection of *p53* and bcl-2 expression in kidney tissue

Through the sequential application of a particular antibody to the antigen (primary antibody), a secondary antibody to the primary antibody, and an enzyme complex with a chromogenic substrate with interposed washing steps, the immunohistochemical application provides the visualisation of antigens. This chromogenic enzyme technique produces such a noticeable reaction at the antigen location. After that, the specimen was over-slipped and counterstained. A light microscope is used to analyze the findings [66, 67]. According to Hsu and Raine [68] and Elias et al. [69], the staining technique for *p*53 and bcl-2 was carried out as follows: Kidney tissue slices were cut and mounted on positive slides covered with an appropriate tissue adhesive. After the tissue sections were rehydrated using graded ethyl alcohols, the sections were de-paraffinized in xylene. Slides were immersed in the peroxidase solution for 10 min after endogenous peroxidase was neutralised using 1 mL of 30% hydrogen peroxide to 9 mL of 100% methanol. Phosphate buffer solution (PBS) (PBS; 120 mM/L NaCl, 11.5 mM/L NaH_2_PO_4_, 31.3 mM/L KH_2_PO_4_, pH 7.4) was used to wash the slides for 2 min. Each slide received 100 µL of primary antibody (clone PC10 which is a mouse monoclonal antibody) (was purchased from Sigma-Aldrich in the United States where the catalogue number: CBL407 and the Trade name: Chemicon), which completely coated the kidney tissue. The slides were then incubated for 30 min before being washed with PBS for two minutes. Each slide received a second secondary antibody (Biotinylated horse antimouse/-rabbit immunoglobulin-G (IgG) secondary antibody) (100 µL), (Biotinylated horse anti-mouse/-rabbit IgG secondary antibody was purchased from Zymed laboratories, Incorporation, San Francisco in the United States) which completely covered the tissue. The slides were then incubated for 20 min before being washed with PBS for three minutes. Each slide received an additional 100 µL of enzyme conjugate to completely cover the kidney tissue. The slides were then incubated for 10 min before being thoroughly washed with PBS for 2 min. The slides were counterstained with hematoxylen after being cleaned in water and chromogen was added. After dehydrating and clearing the slides, the sections were examined under a microscope.

### Histopathological investigation

Kidney specimens were prepared for routine embedding in paraffin after being preserved in a 10% formalin solution. For histological analysis, blocks were sectioned at a thickness of 5 µm and stained with hematoxylin and eosin.

### Statistical analysis

The results were presented as mean standard error mean (SEM) in a table. Using the SPSS 13 program, a one-way Analysis of variance (ANOVA) test was used to determine the differences between the various groups. The Fisher least significant difference (FLSD) test was used in the post-hoc analysis comparing the various groups, with significance set at *P* ≤ 0.05.

## Data Availability

All data are available in the manuscript or upon request to the authors.
